# False Reports Credited as Military News

**Published:** 1899-11

**Authors:** 


A Monthly Review of Medicine and Surgery.

*EDITOR:*

WILLIAM WARREN POTTER, M. D.

All communications, whether of a literary or business nature, books for review and exchanges should be addressed to the editor: 284 FRANKLIN STREET, BUFFALO, N. Y.

VOL. XXXIX.—LV. NOVEMBER, 1899. NO. 4.

### FALSE REPORTS CREDITED AS MILITARY NEWS.

TWO former soldiers of the 13th U.S. Infantry who were discharged in Manila and returned to the United States on the transport Newport, arrived at Buffalo a few days ago, and at once hunted up a newspaper reporter and proceeded to give him harrowing tales of neglect, cruelty, lack of medicine and surgeons, and poor food. They also gave it as their opinion that General Otis was not capable of conducting a successful campaign. Briefly, their statements regarding the medical department of the army were to the effect, that amputations were performed without anesthetics; that the surgeons neglected the soldiers; that men died for want of care and medicine; that the food on the transport was poor; that it was worse in Manila; that there was not a medical officer on the transport; that there were no drugs even; that the undertakers embalmed the men who died en route in the same room with the men, and while meals were in progress, and that in embalming, the undertakers cut the dead "across the abdomen," and "threw parts of the body overboard." In substantiation of their statements regarding the lack of medicine and surgeons on board the transport, they relate an incident, wherein an enlisted man "who was an English doctor" wrote a prescription and gave it to the steward, who handed him back a bottle of colored fluid, which the "English doctor" on sight declared to be not the proper mixture; whereupon the steward "confessed" that there were no drugs on board the vessel.

Naturally, the newspapers printed long, sad tales of the horrible cruelty to the soldiers, and made very delicious Atkinsonesque morsels for the "haul down the flag" people to roll under their tongues.

It is hardly necessary, so far as scarcity of drugs is concerned, to call attention to the supplies sent to the Philippine Islands by the

Surgeon-General of the Army, as published in the JOURNAL two months ago. There are officers of the medical corps detailed at San Francisco and Manila, whose sole duty is to inspect and refit each transport before it leaves on a voyage. Each transport has its complement of medical officers and a full supply of drugs. As for any hospital steward in any branch of the service of the United States army, navy or marine hospital corps, receiving and filling a prescription written by an enlisted man, for use by another person, the statement on its face is preposterous. The balance of the medical statements hardly need reference.

There has been in this campaign, as there has been in every other past campaign, and as there will continue to be in other campaigns to come, just such wild statements made by disgruntled soldiers and anti-expansionists and kickers. But, in all the swirl of war time criticism, just or unjust, Surgeon-General Sternberg's department has been above reproach. He has supplied excellent medical service to the men, and the drug supply has been ample. This is shown by the equipment of the volunteer regiments, each of which has been given an outfit sufficient to medicate a brigade--a regimental outfit, which in the civil war would have been a God-send to an army division, and a blessing to an army corps. Sherman said, "war is hell." It remains for some equally terse and epigrammatical gentleman to properly classify these persons who are deliberately lying about medical equipment and surgeons' cruelty and snarling at the flag.

